# Supplementary material for: A chemical toolbox for the study of bromodomains and epigenetic signaling
Source: Nat Commun. 2019 Apr 23;10:1915. doi: 10.1038/s41467-019-09672-2 (PMC6478789; doi:10.1038/s41467-019-09672-2)
Supplement: Supplementary file 1 — Supplementary Information [file 41467_2019_9672_MOESM1_ESM.pdf]

Supplementary information

A Chemical Toolbox for the Study of Bromodomains and Epigenetic Signaling

Knapp and Arrowsmith et al.

Content:

Page 2	Supplementary figure 1
Page 3	Supplementary figure 2
Page 4	Supplementary figure 3
Page 6	Supplementary table 1
Page 12	Supplementary table 2
Page 14	Supplementary table 3
Page 15	Supplementary table 4
Page 16	Supplementary table 5
Page 17	Supplementary figure 4

Supplementary figure 1

CBP30-298

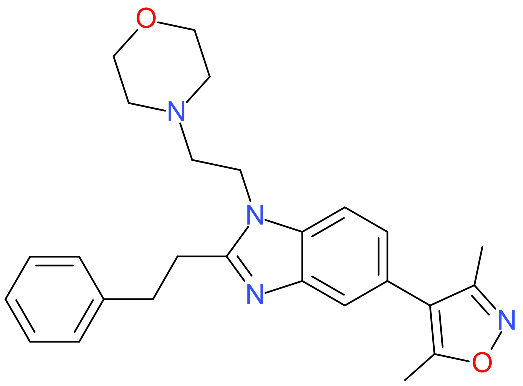

CBP30-383

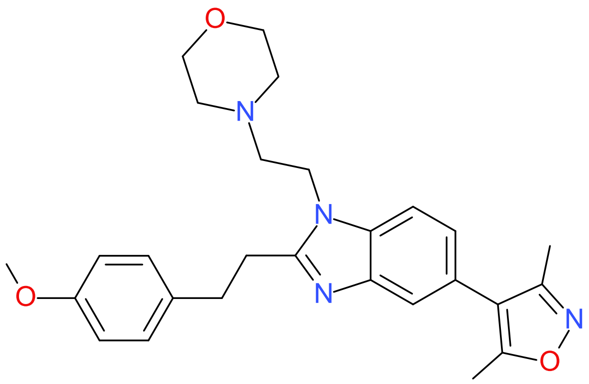

PFI-3 D1

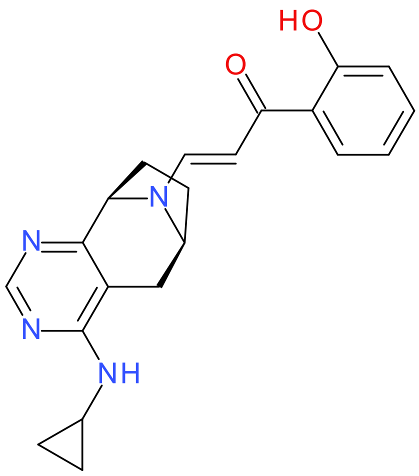

BAY-876

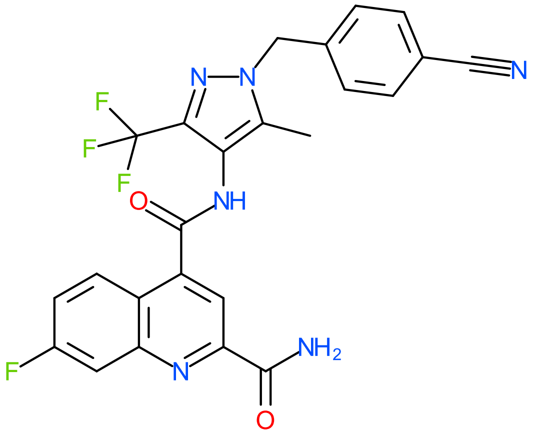

Supplementary figure 1: Chemical Structures of probe related compounds and BAY-876.

Supplementary Figure 2

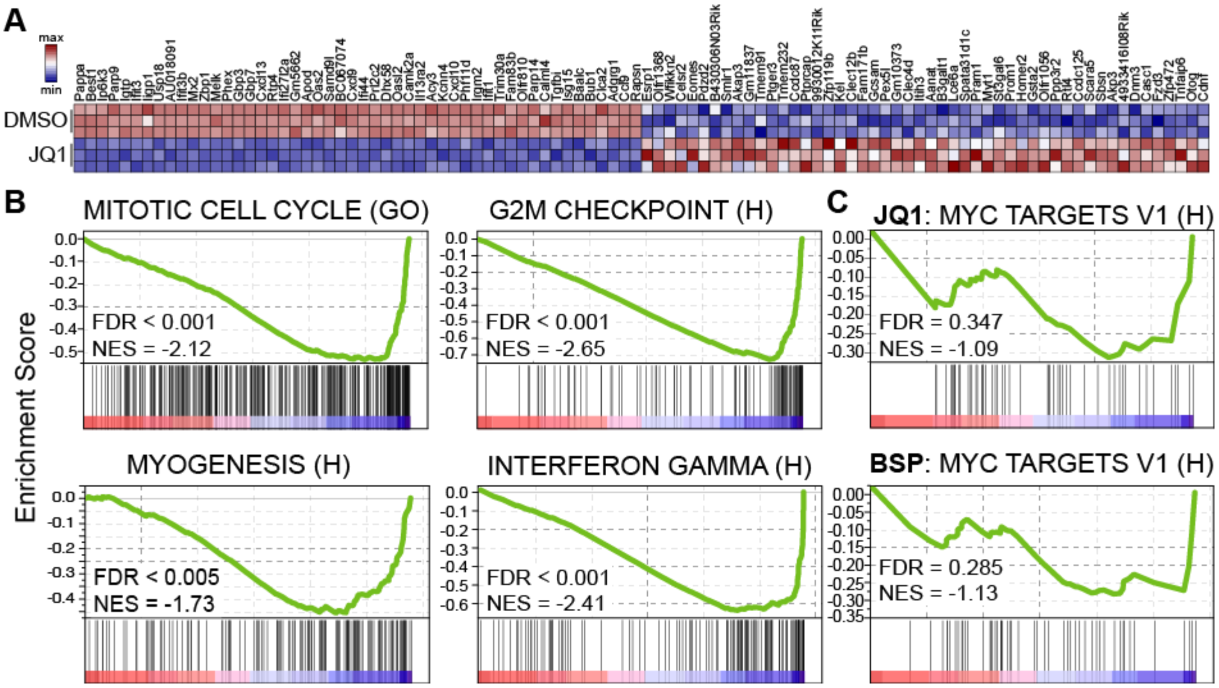

Supplementary Figure 2: Transcriptional response to BSP and JQ1 in C2C12 myoblasts. A) Heatmap of the top 50 up/down regulated genes in C2C12 myoblasts following 12 h JQ1 treatment based on 2-sided signal to noise ratio (SNR) score and *P* < 0.05. Dark blue indicates lowest expression; dark red indicates highest expression, with intermediate values represented by lighter shades, as indicated in the inset. Data are column-normalized. **B)** GSEA demonstrating strong association with mitotic cell cycle (from the Gene Ontology MSigDB set, top left), G2M checkpoint (from hallmark MSigDB signatures, top-right), myogenesis (from hallmark MSigDB signatures, bottom-left) and interferon gamma signalling, following 12 h treatment of C2C12 cells with JQ1. **C)** GSEA demonstrating lack of association with MYC response following 12 h treatment of C2C12 cells with JQ1 (top panel) and BSP (lower panel).

Supplementary Figure 3

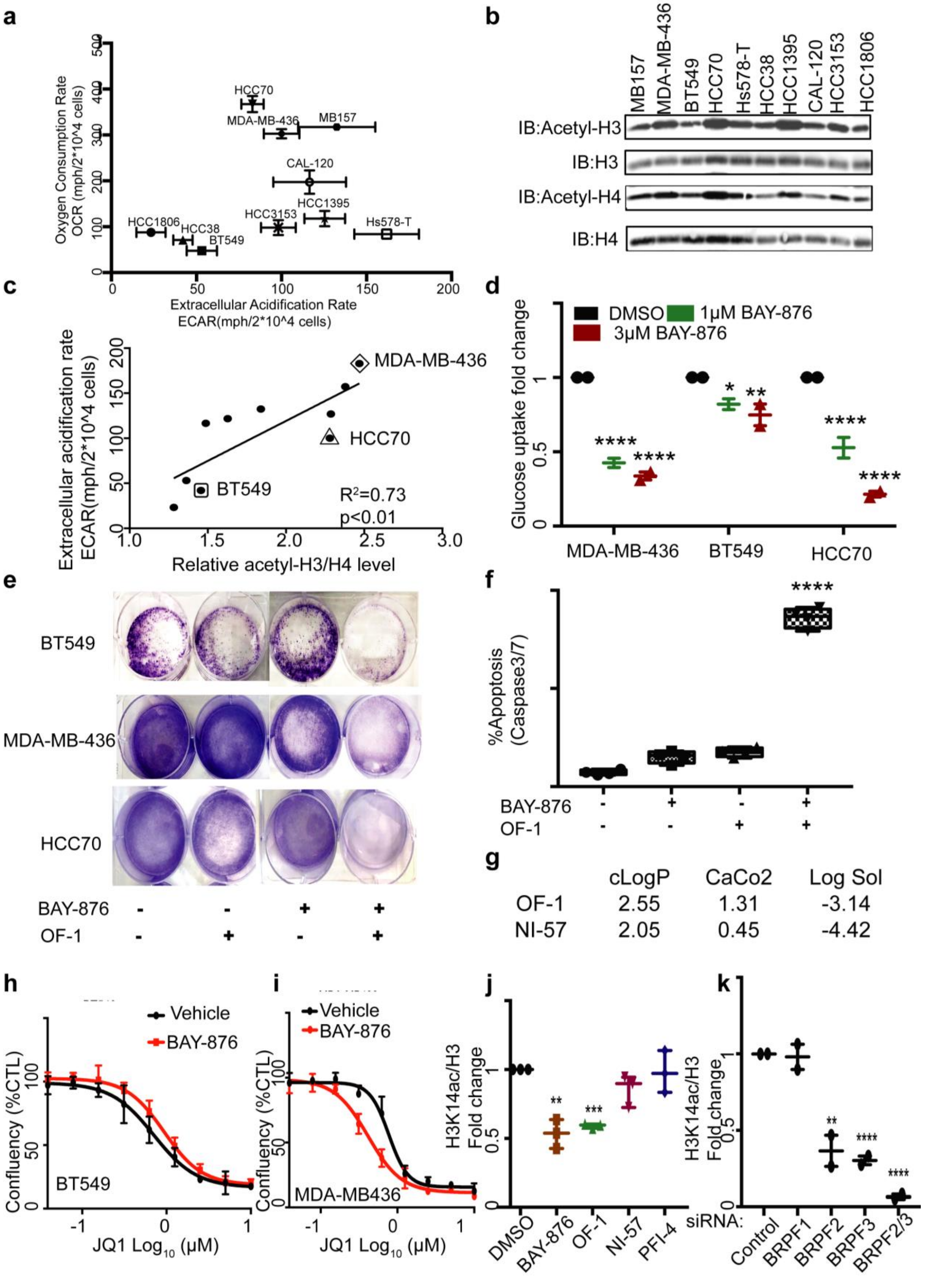

	cLogP	CaCo2	Log Sol
OF-1	2.55	1.31	-3.14
NI-57	2.05	0.45	-4.42

Supplementary Figure 3: Metabolic profiles and synergy with of BRPF bromodomain inhibition with BAY-876 a) Metabolic profiles of ten TNBC lines based on Seahorse XF96 measurements. Depicted are the ECAR (X axis) and the OCR (Y axis) measurements. Graph

indicate mean and error bars denote standard deviation from eight wells (from two independent assays). **b)** A representative immunoblot of histone acetylation level across ten TNBC lines. **c)** Plot of ECAR and histone acetylation across ten TNBC lines. Labeled dots denote the representative TNBC lines, having extreme or medial glycolytic rates and histone acetylation levels, used for the following assays. **d)** Glucose uptake in three representative cell lines in respond to BAY-876 treatment relative to vehicle. Experiment repeated two times. Cells were treated with DMSO or indicated concentration of BAY-876 for 5 days. Graph indicate mean and error bars denote standard deviation from three independent assays. p value computed using a one-way ANOVA test; ****** p<0.001. **e)** The colony formation of indicated cells with 3μM indicated chemical probes treatment for 14 days. **f)** MDA-MB436 cells were treated with 3μM indicated chemical probes for 5 days. Cell apoptotic activity was quantified by the fluorescent caspase 3/7 active objects. Graph indicate mean and error bars denote standard deviation from four independent assays. p value computed using one-way ANOVA t test; **** p<0.001. **g)** Pharmacokinetic properties of NI-57 and OF-1 predicted by pkCSM (http://biosig.unimelb.edu.au/pkcsm/prediction). **h)** Dose dependent curves for cell lines treated with indicated concentrations of JQ-1 with or without 3μM BAY876 for 7 days in BT549 cell line. Graph indicate mean and error bars denote standard deviation from four independent assays. p value computed using a two- stage step-up Benjamini t test **i)** Dose dependent curves for cell lines treated with indicated concentrations of JQ-1 with or without 3μM BAY876 for 7 days in MDA-MB-436 cell line. Graph indicate mean and error bars denote standard deviation from four independent assays. p value computed using a two- stage step-up Benjamini t test **j)** Immunoblot based quantification of H3K14ac abundance using total H3 as a control in MDA-MB-436 cells following control or 3 μM of the indicated chemical probes during a 5-day treatment. Graph indicate mean and error bars denote standard deviation from three independent assays. p value computed using a two-stage step-up Benjamini t test; **p<0.01; *** p<0.001. **k)** Immunoblot based quantification of H3K14ac abundance using total H3 as a control in MDA-MB-436 BRPF knockdown cells. Graph indicate mean and error bars denote standard deviation from two independent assays. p value computed using a two-stage step-up Benjamini t test; **p<0.01; *** p<0.001.

Supplementary Table 1: Probe set for the human bromodomain family

Target	Inhibitor<br>Negative control<br>Supplier‡	SMILE	K_D (nM)	Ref.
**Family I: PCAF/GCN5**	**L-Moses**<br><br>**Negative control: D-Moses**<br><br>**Probe: T**<br>**Control: SGC**	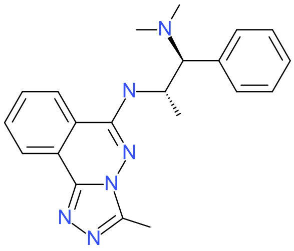	ITC data:<br>126 nM<br>PCAF<br>600 nM<br>GCN5	**1**
**Family I: PCAF/GCN5**	**GSK4027**<br><br>**Negative control: GSK4028**<br>**SGC**	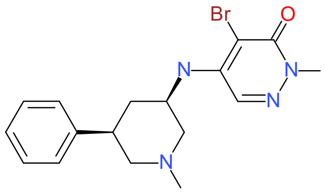	BROMOscan<br>1.4nM PCAF<br>1.4nM GCN5	**2**
**Family I: CECR2**	**NVS-CECR2-1**<br><br>**C, M, T**	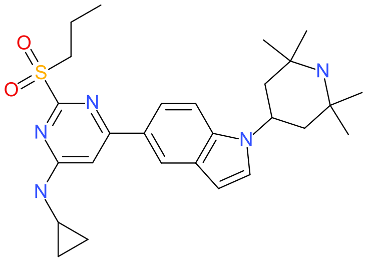	ITC data:<br>80nM CECR2	**3**
**Family I: CECR2**	**GNE-886**<br><br>**MedKoo**	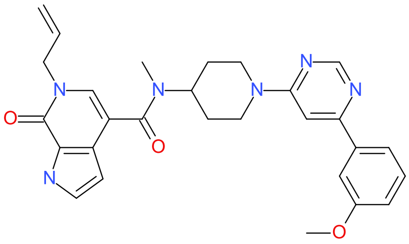	EC_{50}<br>370nM<br>CECR2	**4**
**Family 2 BET (BRD4, 3, 2,T)**	**(+)-JQ1**<br><br>**Negative control: (-)-JQ1**<br><br>**C, M, T**	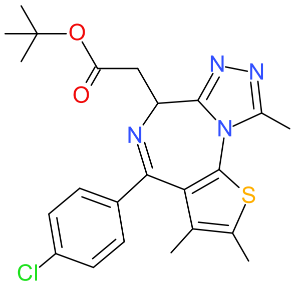	ITC data:<br>49nM<br>BRD4(1)<br>90nM<br>BRD4(2)<br>59nM<br>BRD3(1)<br>82nM<br>BRD3(2)<br>128nM<br>BRD2(1)<br>190nM<br>BRDT(1)	**5**
**Family 2 BET (BRD4, 3, 2,T)**	**I-BET151**<br><br>**C, T**	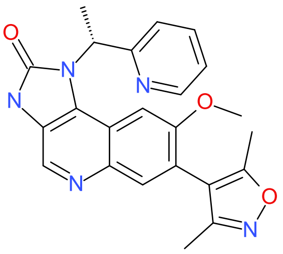	Cell free assay<br>IC50 of 500 nM, 25 nM, and 790nM	**6**

**Family 2<br>BET (BRD4, 3, 2,T)**	**PFI-1**<br><br>**C, M, T**	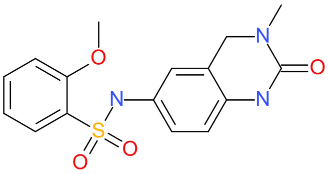	ITC data:<br>47nM<br>BRD4(1)<br>195nM<br>BRD4(2)<br>80nM<br>BRD3(1)<br>76nM<br>BRD3(2)<br>108nM<br>BRD2(1)<br>144nM<br>BRD2(2)<br><br>85nM<br>BRDT(1)<br>221nM<br>BRDT(2)	**7**
**Family III<br>CBP/p300**	**SGC-CBP30**<br><br>**Negative control: BDOIA513**<br><br>**Probe: C, M, T**<br>**Control: SGC**	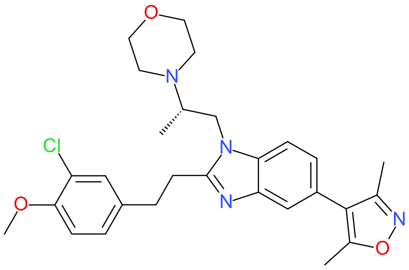	ITC data:<br>21nM CBP<br>38nM p300	**8**
**Family III<br>CBP/p300**	**I-CBP112**<br><br>**C, M, T**	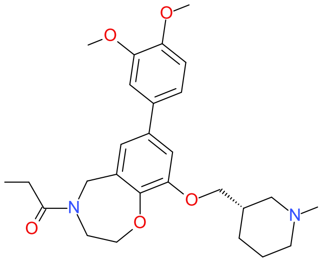	ITC data:<br>151nM CBP<br>625nM p300	**9**
**Family III<br>CBP/p300**	**GNE-781**<br><br>**MedKoo**	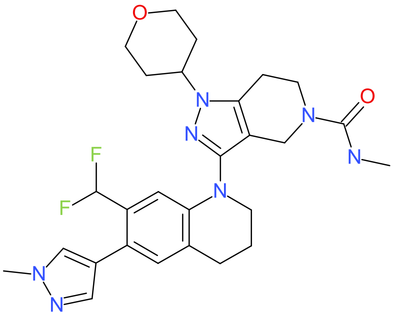	TR-FRET:<br>0.94 nM<br>BRET:<br>6.2 nM	**10**
**Family IV<br>pan-BRPF**	**OF-1**<br><br>**C, M, T**	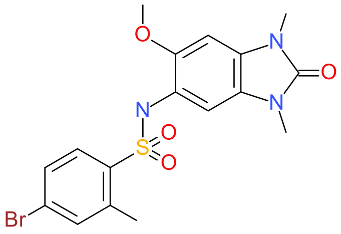	ITC data:<br>101nM<br>BRPF1B<br>505nM<br>BRPF2<br>2400nM<br>BRPF3<br><br>Off target<br>BRD4(1)<br>3900 nM	**11**

**Family IV<br>pan-BRPF**	**GSK6853**<br><br>**Negative control:<br>GSK9311**<br><br>**Probe: C, M<br>Control: M**	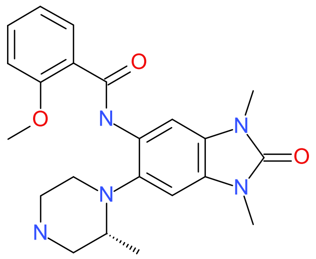	TR-FRET<br>81nM<br>BRPF1B	**12**
**Family IV<br>pan-BRPF**	**NI-57**<br><br>**C, M, T**	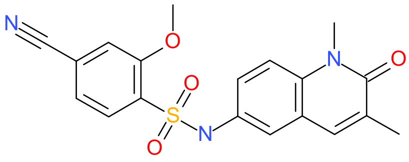	ITC data:<br>31nM<br>BRPF1B<br>108nM<br>BRPF2<br>409nM<br>BRPF3<br>Off target<br>BRD9<br>998nM	**11**
**Family IV<br>BRPF1B**	**PFI-4**<br><br>**C, M, T**	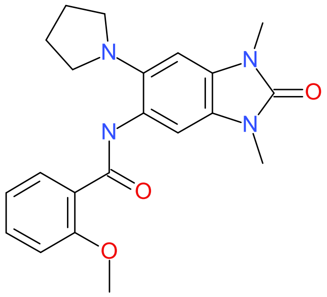	ITC data:<br>13nM<br>BRPF1B<br>775nM<br>BRPF2<br>Off target<br>CECR2<br>2350nM	**11**
**Family IV<br>BRPF2**<br><br>**Family VII<br>TAF1(2)<br>TAf1L(2)**	**BAY-299**<br><br>**Negative control:<br>BAY-364**<br><br>**C, M, T**	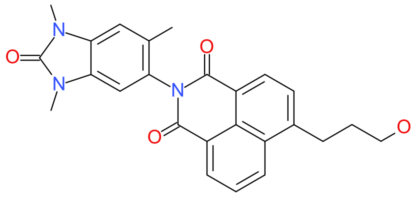	ITC data:<br>44nM BRPF2<br>16nM<br>TAF1(2)<br>234nM<br>TAF1L(2)<br><br>Off target<br>1390nM CBP	**13**
**Family IV<br>BRD7/BRD9**	**TP-472**<br><br>**Negative control:<br>TP-472N**<br><br>**C, M, T**	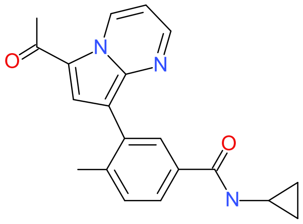	ITC data:<br>33nM BRD9<br>340nM BRD7	**14**
**Family IV<br>BRD7/BRD9**	**BI-9564**<br><br>**C, M, T**	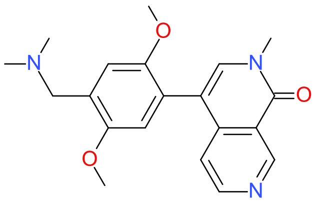	ITC data:<br>15nM BRD9<br>BROMOscan<br>0.75nM<br>BRD9<br>0.3 nM BRD7<br>Off target<br>ITC<br>CECR2:<br>187nM	**15**

**Family IV<br>BRD7/BRD9**	**LP-99**<br><br>**Negative control: (2S,3R)-LP99**<br><br>**Probe: C, M, T**<br>**Control: SGC**	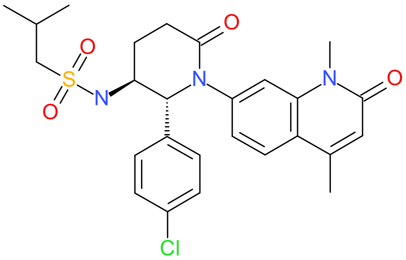	ITC data:<br>99nM BRD9<br>909nM BRD7	**16**
**Family IV<br>BRD7/BRD9**	**I-BRD9**<br><br>**C, M, T**	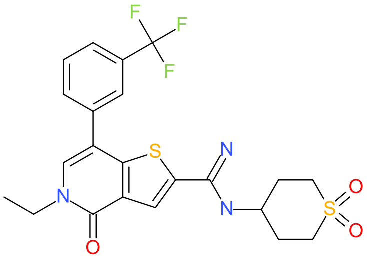	BROMOscan:<br>1.9 nM BRD9<br>380nM BRD7<br><br>Off target<br>CECR2<br>140nM<br>CBP 740nM<br>p300 770nM<br>BRPF1<br>2100nM	**17**
**(BRPF1B)<br>(ATAD2<br>8Family IV<br>ATAD2B**	**GSK8814**<br><br>**Negative control: GSK8815**<br><br>**SGC**	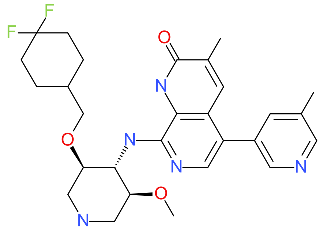	ITC<br>10 nM<br>ATAD2B	**18**
**Family IV<br>and VI<br>TRIM24<br>BRPF1**	**Benzimidazolone 8**<br><br>**SGC**	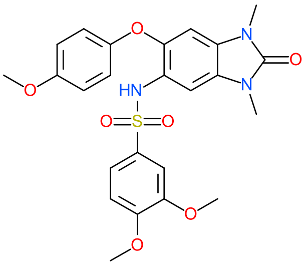	ITC<br>220nM<br>TRIM24<br>140nM<br>BRPF1B<br>1240nM<br>BRPF2	**19**
**Family IV<br>ATAD2A**	**BAY-850**<br><br>**Negative control: BAY-460**<br><br>**SGC**	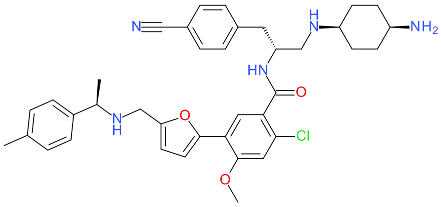	BROMOscan<br><br>120nM<br>ATAD2A	**20**
**Family VI<br>BAZ2A/B**	**BAZ2-ICR**<br><br>**C, M, T**	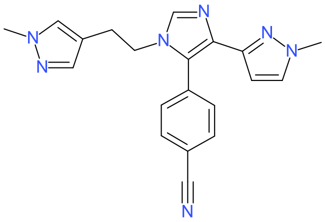	ITC:<br>109nM<br>BAZ2A<br>170nM<br>BAZ2B<br><br>Off-target<br>1550nM<br>CECR2	**21**
**Family VI<br>BAZ2A/B**	**GSK2801**<br><br>**Negative control: GSK8573**<br><br>**Probe: C, M, T**	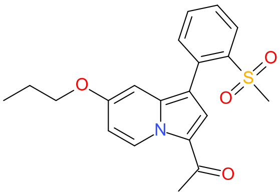	ITC:<br>257nM<br>BAZ2A<br>136nM<br>BAZ2B	**22**

**Family VIII SMARCA2/4 PB1(5)**	**PFI-3**<br><br>**Negative control: BDF25488524**<br><br>**Probe: C, M, T**<br>**Control: SGC**	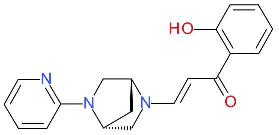	ITC:<br>89nM SMARCA4<br>48nM PB1 (5)	**23**

‡Cayman Chemical (C), Millipore-Sigma (M), Tocris (T)

References:

1. Moustakim, M. et al. Discovery of a PCAF Bromodomain Chemical Probe. *Angew Chem Int Ed Engl* **56**, 827-831 (2017).
2. Humphreys, P.G. et al. Discovery of a Potent, Cell Penetrant, and Selective p300/CBP-Associated Factor (PCAF)/General Control Nonderepressible 5 (GCN5) Bromodomain Chemical Probe. *J Med Chem* **60**, 695-709 (2017).
3. SGC. NVS-CECR2-1. https://www.thesgc.org/chemical-probes/NVS-1 (2015).
4. Crawford, T.D. et al. GNE-886: A Potent and Selective Inhibitor of the Cat Eye Syndrome Chromosome Region Candidate 2 Bromodomain (CECR2). *ACS Med Chem Lett* **8**, 737-741 (2017).
5. Filippakopoulos, P. et al. Selective inhibition of BET bromodomains. *Nature* **468**, 1067-73 (2010).
6. Mirguet, O. et al. From ApoA1 upregulation to BET family bromodomain inhibition: discovery of I-BET151. *Bioorg Med Chem Lett* **22**, 2963-7 (2012).
7. Picaud, S. et al. PFI-1, a highly selective protein interaction inhibitor, targeting BET Bromodomains. *Cancer Res* **73**, 3336-46 (2013).
8. Hay, D.A. et al. Discovery and optimization of small-molecule ligands for the CBP/p300 bromodomains. *J Am Chem Soc* **136**, 9308-19 (2014).
9. Picaud, S. et al. Generation of a Selective Small Molecule Inhibitor of the CBP/p300 Bromodomain for Leukemia Therapy. *Cancer Res* **75**, 5106-5119 (2015).
10. Romero, F.A. et al. GNE-781, A Highly Advanced Potent and Selective Bromodomain Inhibitor of Cyclic Adenosine Monophosphate Response Element Binding Protein, Binding Protein (CBP). *J Med Chem* **60**, 9162-9183 (2017).
11. Meier, J.C. et al. Selective Targeting of Bromodomains of the Bromodomain-PHD Fingers Family Impairs Osteoclast Differentiation. *ACS Chem Biol* **12**, 2619-2630 (2017).
12. Bamborough, P. et al. GSK6853, a Chemical Probe for Inhibition of the BRPF1 Bromodomain. *ACS Med Chem Lett* **7**, 552-7 (2016).
13. Bouche, L. et al. Benzoisoquinolinediones as Potent and Selective Inhibitors of BRPF2 and TAF1/TAF1L Bromodomains. *J Med Chem* **60**, 4002-4022 (2017).
14. SGC. TP-472. https://www.thesgc.org/chemical-probes/TP-472 (2016).
15. Martin, L.J. et al. Structure-Based Design of an in Vivo Active Selective BRD9 Inhibitor. *J Med Chem* **59**, 4462-75 (2016).
16. Clark, P.G. et al. LP99: Discovery and Synthesis of the First Selective BRD7/9 Bromodomain Inhibitor. *Angew Chem Int Ed Engl* **54**, 6217-21 (2015).
17. Theodoulou, N.H. et al. Discovery of I-BRD9, a Selective Cell Active Chemical Probe for Bromodomain Containing Protein 9 Inhibition. *J Med Chem* **59**, 1425-39 (2016).
18. Bamborough, P. et al. A Chemical Probe for the ATAD2 Bromodomain. *Angew Chem Int Ed Engl* **55**, 11382-6 (2016).
19. Bennett, J. et al. Discovery of a Chemical Tool Inhibitor Targeting the Bromodomains of TRIM24 and BRPF. *J Med Chem* **59**, 1642-7 (2016).
20. Fernandez-Montalvan, A.E. et al. Isoform-Selective ATAD2 Chemical Probe with Novel Chemical Structure and Unusual Mode of Action. *ACS Chem Biol* **12**, 2730-2736 (2017).
21. Drouin, L. et al. Structure enabled design of BAZ2-ICR, a chemical probe targeting the bromodomains of BAZ2A and BAZ2B. *J Med Chem* **58**, 2553-9 (2015).

22. Chen, P. et al. Discovery and Characterization of GSK2801, a Selective Chemical Probe for the Bromodomains BAZ2A and BAZ2B. *J Med Chem* **59**, 1410-24 (2016).
23. Fedorov, O. et al. Selective targeting of the BRG/PB1 bromodomains impairs embryonic and trophoblast stem cell maintenance. *Sci Adv* **1**, e1500723 (2015).

Supplementary Table 2

Compound	TTK	CK2A1	CAMK1G	AAK1	ABL1	AURKB	BRAF	CAMKK2	STK10
L-Moses	-0.5	-0.1	-0.3	-1.6	0.0	0.4	-0.1	-0.1	-0.4
I-CBP112	0.0	-1.9	-0.2	-0.7	0.0	0.3	-0.1	-0.4	-0.4
PFI-4	-0.6	-0.5	-0.6	-0.9	0.0	0.4	0.6	0.4	-0.4
LP-99	-0.1	-1.4	-0.2	-0.8	-0.1	0.1	-0.2	0.0	-0.3
PFI-3	-0.7	0.3	0.0	-0.3	-0.1	0.2	-0.1	-0.1	-0.2
NVS-CECR2-1	0.4	-1.0	-0.4	-0.7	0.0	0.3	-0.1	-0.3	-0.1
OF-1	0.1	-2.4	-0.7	-0.9	-0.1	0.3	0.0	-0.3	-0.2
BAY-299	-0.1	-1.4	-0.6	-0.4	0.0	0.2	0.1	-0.4	-0.2
I-BRD9	0.2	-1.1	-0.3	-0.4	0.0	0.2	0.0	-0.2	-0.2
JQ1	-0.5	-1.4	-0.9	-2.8	-0.2	-0.1	-0.4	-0.6	-0.6
GSK6853	0.1	-0.7	-0.5	-0.8	0.1	1.0	0.1	0.5	0.1
TP-472	0.2	-1.1	-0.2	-0.3	0.1	0.2	0.8	0.0	-0.2
BAZ2-ICR	0.3	-1.3	-0.1	0.8	0.0	0.0	0.1	0.0	0.0
SGC-CBP30	-0.4	-1.8	-0.6	-0.6	-0.2	0.0	-0.5	-0.6	0.0
NI-57	0.0	-1.7	-0.6	0.2	-0.1	0.0	0.0	-0.4	0.2
BI-9564	0.1	-1.2	-0.6	0.1	0.0	0.1	-0.2	-0.2	-0.2
GSK2801	0.4	-1.0	-0.3	0.5	0.0	-0.1	-0.3	0.1	-0.5

Compound	EPHA2	FES	GPRK5	GSG2	MERTK	PAK4	MAPK1	MAPK8	STK3
L-Moses	1.1	-0.2	-0.3	0.1	0.0	-0.3	0.1	0.6	0.0
I-CBP112	1.1	-0.1	-0.4	0.1	-0.2	-0.2	0.2	0.3	0.0
PFI-4	1.8	0.0	-0.3	0.3	-0.1	-0.2	0.2	-0.2	0.0
LP-99	0.9	0.0	-0.3	-0.2	-0.2	-0.4	0.2	-0.1	-0.2
PFI-3	-0.2	-0.2	-0.2	0.0	-0.1	-0.2	-0.1	0.1	-0.1
NVS-CECR2-1	1.5	-0.5	-0.1	0.2	0.0	-0.1	0.3	-0.3	-0.1
OF-1	1.2	-0.6	-0.4	0.2	-0.1	-0.2	0.3	-0.1	0.0
BAY-299	2.0	0.0	-0.3	0.1	-0.2	-0.1	0.0	-0.4	0.1
I-BRD9	1.2	0.1	-0.1	0.1	0.0	-0.1	0.1	-0.1	0.1
JQ1	0.9	-0.8	0.1	0.1	-0.3	-0.4	0.2	-0.8	0.1
GSK6853	1.5	-0.2	-0.3	0.6	0.3	0.1	0.2	0.1	0.3
TP-472	2.1	0.0	-0.1	0.1	-0.1	0.0	0.2	0.2	0.1
BAZ2-ICR	1.1	0.0	0.0	0.0	0.0	0.0	0.1	0.2	0.0
SGC-CBP30	1.2	-0.6	0.1	0.0	-0.1	0.0	0.2	0.8	0.0
NI-57	1.5	-0.4	-0.3	0.0	0.0	0.1	0.1	0.1	0.2
BI-9564	2.1	-0.1	-0.4	0.0	0.2	0.0	0.1	0.0	0.0
GSK2801	1.2	-0.2	-0.5	0.2	0.0	-0.2	0.2	0.1	0.1

Compound	CASK	BMX	CSNK1D	DAPK3	DYRK1A	PHGK2	PIM1	RPS6KA1
L-Moses	-0.1	-0.1	0.6	0.2	0.2	-0.1	2.3	0.2
I-CBP112	0.0	-0.1	0.5	0.0	-0.4	0.1	-0.1	0.1
PFI-4	-0.2	0.6	1.3	0.4	-0.5	0.1	0.5	0.1
LP-99	-0.3	0.2	0.2	-0.1	-0.3	-0.1	-0.2	-0.1
PFI-3	-0.2	0.3	0.3	0.0	-0.1	0.1	0.1	-0.2
NVS-CECR2-1	0.1	0.0	0.4	0.7	-0.3	-0.2	0.4	0.2
OF-1	0.0	0.5	0.6	-0.1	-0.8	0.1	-0.2	0.0
BAY-299	-0.1	0.3	1.2	-0.2	-0.5	0.0	-0.2	0.1
I-BRD9	-0.2	0.3	0.0	-0.2	-0.6	0.0	0.1	0.1
JQ1	0.1	-0.3	0.4	-0.1	0.2	-0.3	-0.3	-0.1

GSK6853	0.1	0.3	1.3	1.1	-0.2	0.2	3.7	0.2
TP-472	-0.1	0.5	1.1	0.0	-0.3	0.1	0.4	0.1
BAZ2-ICR	0.2	0.5	0.4	-0.1	-0.1	-0.1	0.2	0.1
SGC-CBP30	0.5	-0.2	0.3	0.2	-0.2	-0.3	-0.2	0.0
NI-57	0.3	0.0	0.6	0.0	0.1	0.1	0.1	0.2
BI-9564	0.0	0.1	1.1	0.1	0.1	-0.1	0.0	0.1
GSK2801	0.5	0.2	0.2	-0.2	0.4	-0.2	-0.3	-0.1

Supplementary table 2: Selectivity screening data (protein kinases): Temperature shift data measured on protein kinases. Shown are average data in Kelvin of three measurements. No significant Tm shifts have been observed.

Supplementary Table 3

Probes	ΔTm (°C) 10µM									
	UHRF1	RBBP1	53BP1	BRPF1	BRPF3	WDR5	EED	TDRD3	FXR1	SND1
BAY-299	NA	-1.0	0.3	-2.5	1.5	0.2	NA	-1.4	0.8	NA
BAY-588	-0.4	-0.1	-2.3	-0.2	NA	-7.3	-0.5	-5.3	-1.2	-1.0
BAY-850	-0.3	-1.0	-0.7	-2.2	-0.4	0.2	NA	-1.8	-1.7	-1.6
BAY-876	-0.2	-0.3	0.0	-0.3	-0.3	0.4	-0.7	-1.0	0.8	-0.9
BI-9564	-0.2	0.0	-0.3	-0.3	-0.6	-0.2	-0.7	-0.6	0.3	-0.9
GSK4027	-0.2	-0.7	-0.1	-0.8	-0.3	0.2	-0.7	-0.6	0.3	-1.0
GSK6853	-0.2	-0.7	-0.1	-0.9	-0.4	0.0	-0.9	-0.7	-1.1	-0.6
GSK8814	-0.2	-0.1	-0.1	-0.2	-0.2	0.2	-0.4	-0.6	0.4	-0.5
I-BRD9	-0.1	0.0	0.1	0.2	0.1	0.1	-0.7	-0.4	0.1	-0.3
I-CBP112	0.0	-0.1	0.3	-0.2	0.0	-0.2	-0.5	-0.6	0.9	-0.7
JQ1	-0.2	-0.7	-0.4	-0.6	-0.7	-0.3	-0.9	-1.4	-0.5	-0.6
L-Moses	NA	-0.4	NA	-2.7	1.0	0.5	-0.3	-0.9	0.3	-1.0
LP99	NA	-0.7	0.3	-2.0	0.0	0.4	-0.2	-0.5	0.1	-0.4
NI57	NA	-0.1	-0.1	NA	-0.4	0.3	-0.4	-0.7	-0.5	-0.4
NVS-CECR2-1	-0.3	-1.0	-0.6	-0.2	-0.3	-0.2	-1.0	-3.3	-5.1	-1.7
OF-1	0.2	-0.7	-0.1	-0.6	0.0	-0.2	-0.7	-1.0	0.7	-0.7
PFI-1	0.2	-0.6	-0.2	-0.3	-0.2	0.0	-0.9	-1.7	-0.1	-0.7
PFI-3	0.5	-0.7	0.1	-0.5	0.4	0.2	-0.9	-0.6	0.4	-0.6
PFI-4	-0.5	-1.2	-0.7	-0.7	0.0	-0.5	-2.2	-1.5	-1.2	-1.1
SGC-CBP30	0.0	-1.2	-0.2	0.6	0.2	0.2	-0.4	-1.1	-1.2	-0.4
TP-238	0.0	-0.4	0.4	-0.3	0.2	0.2	-0.3	-0.7	0.4	-0.5
TP-472	-0.1	-0.5	0.1	0.2	0.3	0.0	-0.5	-0.1	0.8	-0.5

Supplementary Table 3: Selectivity screening data (non-BRD reader domains): Temperature shift assay data of BRD probes screened against a diverse panel of methyl-lysine reader domains. Compounds were screened a concentration of 10 µM. Shown are temperature shifts in Kelvin. No significant Tm shifts have been observed.

Supplementary Table 4

Probes	HATs Activity (%) at 1 and 10 µM									
	EP300		HAT1		MYST1		MYST3		GCN5L2	
	1µM	10µM	1µM	10µM	1µM	10µM	1µM	10µM	1µM	10µM
**BAY-299**	95	93	100	93	99	99	98	85	99	103
**BAY-588**	103	99	99	97	100	93	99	96	94	95
**BAY-850**	98	87	101	101	93	94	96	97	99	102
**BAY-876**	99	95	97	89	96	91	99	101	100	97
**BI-9564**	99	94	99	96	106	101	99	100	102	100
**GSK4027**	93	90	102	89	102	98	101	101	99	102
**GSK6853**	98	104	100	94	99	101	107	104	107	107
**GSK8814**	96	102	100	95	99	103	96	102	102	100
**I-BRD9**	96	99	103	101	104	104	101	99	104	102
**I-CBP112**	97	99	100	94	98	103	98	99	105	102
**JQ1**	99	102	98	103	99	95	108	100	104	98
**L-Moses**	94	90	100	95	97	96	92	90	99	95
**LP99**	99	98	104	95	90	88	96	100	92	89
**NI57**	94	93	104	104	96	91	98	99	100	96
**NVS-CECR2-1**	90	84	99	88	96	94	98	103	100	100
**OF-1**	100	103	92	93	102	94	100	102	98	93
**PFI-1**	99	100	102	93	101	95	105	86	96	93
**PFI-3**	98	97	96	94	99	99	95	96	99	101
**PFI-4**	99	101	98	96	98	101	97	102	98	96
**SGC-CBP30**	98	92	96	97	103	97	103	103	101	101
**TP-238**	99	95	93	100	99	97	97	99	96	92
**TP-472**	95	96	98	97	100	103	99	98	94	92

Supplementary Table 4: Selectivity screening data (HATs): Effects of BRD probe compounds on the activity of histone acetyltransferases (HATs). Shown are inhibition data (% inhibition) at a compound concentration of 1 µM and 10 µM.

Supplementary Table 5

MTases	MTases Inhibition (%) at 10µM							
	BAY-588	BAY-850	BAY-876	GSK4027	GSK6853	GSK8814	L-Moses	TP-238
ASH1L	-6	3	4	13	4	2	-3	-4
G9A	3	7	-3	1	-2	-2	-3	7
GLP	2	2	0	2	3	2	2	1
MLL1 (Trimeric)	7	-1	11	8	7	6	20	17
MLL3 (pentameric)	7	0	0	2	2	0	1	-3
NSD2	2	-1	0	15	13	24	2	3
NSD1	11	2	2	4	1	19	-6	1
NSD3	-2	14	3	10	7	13	5	15
PRC2-EZH1	3	19	-4	-1	1	1	5	6
PRC2-EZH2	3	3	5	-5	0	2	-4	3
PRDM9	3	10	0	3	-2	0	4	-1
SETD2	0	7	-4	-1	1	7	11	19
SETD7	10	11	10	12	7	7	7	-1
SETD8	-3	2	0	10	-6	-7	-8	-6
SETDB1	5	8	-4	-1	1	1	1	-1
SMYD2	8	19	-10	2	-3	-2	-10	-2
SMYD3	7	6	3	1	9	8	10	7
SUV39H1	12	5	16	-3	-3	-2	-2	-1
SUV39H2	12	-2	-2	5	1	3	5	7
SUV420H1	-5	5	-1	8	5	-3	4	3
SUV420H2	-3	0	-1	-1	-2	0	6	0
DOT1L	3	3	-6	-2	10	5	1	6
PRMT1	0	15	2	4	-7	3	-2	0
PRMT3	5	3	1	2	1	3	0	5
PRMT4	9	25	1	-2	2	1	6	3
PRMT5	4	4	-1	8	3	-2	-3	-12
PRMT6	4	13	-3	6	5	0	5	6
PRMT7	13	3	12	15	11	11	8	9
PRMT8	3	4	9	15	3	5	5	3
PRMT9	3	15	12	8	12	3	13	7
BCDIN3D	16	28	17	14	5	7	9	4
DNMT1	9	4	4	3	-8	-3	3	3
DNMT3A/3L	-1	21	6	3	13	8	2	8
DNMT3B/3L	-6	28	1	1	8	18	-1	0

Supplementary table 5: Selectivity screening data measured against a panel of lysine methyl transferases (MTases). Inhibition (%) was assess at inhibitor concentration of 10 µM. Shown are average values of two measurements.

Supplementary Figure 4

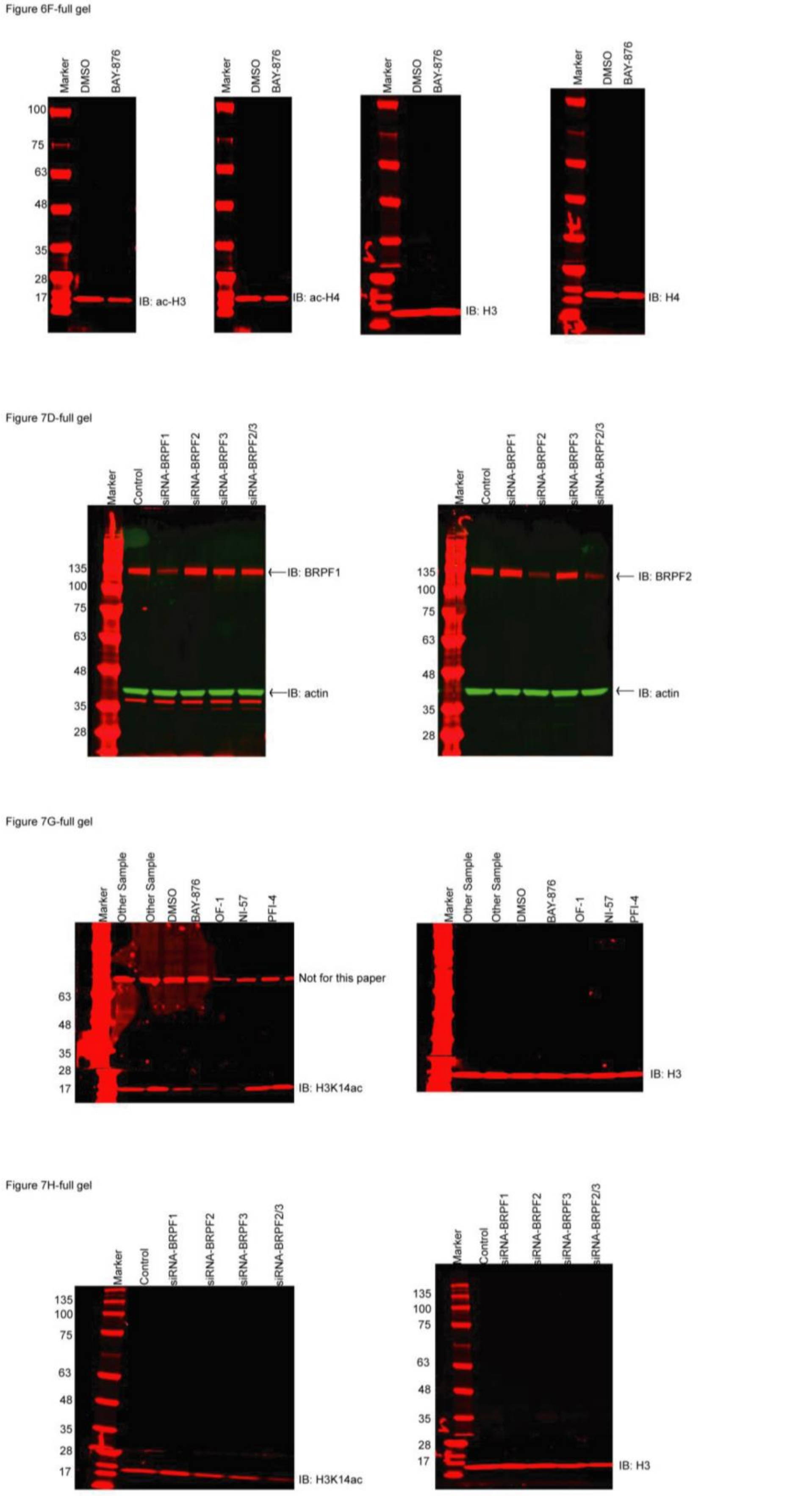

Supplementary Figure 4: Raw data blots used in figure 6 and 7.
