# Supplementary material for: A chemical toolbox for the study of bromodomains and epigenetic signaling
Source: Nat Commun. 2019 Apr 23;10:1915. doi: 10.1038/s41467-019-09672-2 (PMC6478789; doi:10.1038/s41467-019-09672-2)
Supplement: Supplementary file 3 — Description of Additional Supplementary Files [file 41467_2019_9672_MOESM3_ESM.docx]

**Description of Additional Supplementary Files**

File Name: Supplementary Data 1

Description: Bromoscan data summarizing the determined Kd values, the standard deviation for these values as well as structural information (SMILES) for each bromodomain probe tested.
